# Expression of Concern: LncRNA MALAT1 up-regulates VEGF-A and ANGPT2 to promote angiogenesis in brain microvascular endothelial cells against oxygen-glucose deprivation via targetting miR-145

**DOI:** 10.1042/BSR-20180226_EOC

**Published:** 2020-06-24

**Keywords:** Stroke, BMECs, lncRNA-MALAT1

The Editorial Office has been made aware of potential issues surrounding the scientific validity of this paper, hence has issued an expression of concern to notify readers whilst the Editorial Office investigates.

